# Contextual Change After Fear Acquisition Affects Conditioned Responding and the Time Course of Extinction Learning—Implications for Renewal Research

**DOI:** 10.3389/fnbeh.2015.00337

**Published:** 2015-12-08

**Authors:** Rachel Sjouwerman, Johanna Niehaus, Tina B. Lonsdorf

**Affiliations:** Department of Systems Neuroscience, University Hospital Hamburg-EppendorfHamburg, Germany

**Keywords:** context, skin conductance response (SCR), subjective fear ratings, fear potentiated startle, fear conditioning, fear extinction

## Abstract

Context plays a central role in retrieving (fear) memories. Accordingly, context manipulations are inherent to most return of fear (ROF) paradigms (in particular renewal), involving contextual changes *after* fear extinction. Context changes are, however, also often embedded during earlier stages of ROF experiments such as context changes between fear acquisition and extinction (e.g., in ABC and ABA renewal). Previous studies using these paradigms have however focused exclusively on the context switch after extinction (i.e., renewal). Thus, the possibility of a general effect of context switch on conditioned responding that may not be conditional to preceding extinction learning remains unstudied. Hence, the current study investigated the impact of a context switch between fear acquisition and extinction on immediate conditioned responding and on the time-course of extinction learning by using a multimodal approach. A group that underwent contextual change after fear conditioning (AB; *n* = 36) was compared with a group without a contextual change from acquisition to extinction (AA; *n* = 149), while measuring physiological (skin conductance and fear potentiated startle) measures and subjective fear ratings. Contextual change between fear acquisition and extinction had a pronounced effect on both immediate conditioned responding and on the time course of extinction learning in skin conductance responses and subjective fear ratings. This may have important implications for the mechanisms underlying and the interpretation of the renewal effect (i.e., contextual switch *after* extinction). Consequently, future studies should incorporate designs and statistical tests that disentangle general effects of contextual change from genuine ROF effects.

## Introduction

In our daily lives events are usually embedded in a broader set of circumstances (i.e., context). These contexts do not only frame the perception and interpretation of an event but also guide what is later remembered. In addition, contexts function as retrieval cues and thus ultimately gate behavioral responses. The definition however, of what constitutes a context is complex. Generally, the physical environment (i.e., the combination of internal and external states) is considered to constitute the context (Maren et al., 2013). Importantly, patients suffering from anxiety and stress-related disorders often fail to respond appropriately in clearly innocuous situations, which can be distinguished from dangerous situations through contextual signals. For instance, patients suffering from spider phobia might drop their plate of hot food even when seeing a spider on television or post-traumatic stress disorder patients might jump to the floor looking for shelter after hearing a smashing door.

The acquisition of such fearful behavior can be modeled in the laboratory in classical conditioning paradigms where a neutral cue (conditioned stimulus, CS+), such as a geometric figure, acquires the capacity to predict an aversive event (unconditioned stimulus, US), such as an electrotactile stimulus. After conditioning, the CS+ elicits a conditioned response (CR), which can be measured through physiological responding (e.g., skin conductance response, fear potentiated startle) while a neutral cue that is not predictive of the US (CS−) generally does not. A waning of the conditioned response (i.e., extinction) can be achieved through presentations of the CS+ without being followed by the US. Thereby, extinction does not lead to erasure of the initial CS-US memory but induces new inhibitory (safety) learning (for a review see Milad and Quirk, 2012). This is made evident from return of fear (ROF) phenomena (Bouton, 2002) such as ROF after the mere passage of time (spontaneous recovery, SR), un-signaled US presentations (reinstatement, RI), and contextual changes (renewal, RN) after successful extinction. Critically, these ROF phenomena are context dependent, involving changes in the temporal (SR) or physical context (RN) as well as involving context conditioning (RI).

Importantly, the context in which extinction, or in clinical terms, treatment of anxiety disorders, takes place (context B) is nearly always different from the context in which fear was originally acquired (context A). This is of critical importance, as the context gates which memory type (CS-US vs. CS-noUS) is eventually expressed when confronted with ambiguous cues (Bouton, 1993; Maren et al., 2013). Presenting acquisition and extinction in different contexts in the laboratory is thought to disambiguate the CS-US association from the CS-noUS association through learning that under certain contextual circumstances the CS-US association is not valid (occasion setting). Furthermore, different contexts during acquisition and extinction allow for the investigation of extinction without the confounding effects of the fear–inducing acquisition context, which boosts fearful responding also in the absence of the CS+.

Consequently, a context change from acquisition (in context A) to extinction (in context B) is common in experimental designs. Critically, the most frequently used paradigms in renewal research (ABA and ABC renewal) involve a context switch after both acquisition and extinction (Vervliet et al., 2013). In rodents, AAB renewal with conditioning and extinction taking place in the same context (A) has also been described, but it is not as robust as ABA and ABC renewal (Bouton and King, 1983). Per definition, the main focus of renewal studies is the context change *after* extinction (i.e., renewal), which is common to all three paradigms (ABA, ABC, AAB). In contrast, the possible impact of a context change after acquisition (i.e., in ABA or ABC but not in AAB renewal) has not received much attention to date. However, if a context switch following acquisition affects conditioned responding already *during* extinction, this might have important implications for the possible mechanisms underlying renewal effects induced by a context change *following* extinction. More precisely, if contextual change exerts a *general* effect on conditioned responding that is not pertinent to already occurred extinction learning, this may challenge the interpretation of the mechanisms thought to underlie renewal.

Indeed preliminary evidence for an effect of contextual change following acquisition on early extinction performance can be derived from the renewal literature. However, firm conclusions are precluded due to the selective focus on renewal in both study design (i.e., ABA/ABC paradigms without AAA/AAC control groups) and statistical analyses. Three studies report longer response times for US expectancy ratings to both CSs (Neumann and Kitlertsirivatana, 2010; Bandarian Balooch and Neumann, 2011) and a decrease in CS-discrimination (Effting and Kindt, 2007) on the first extinction trial after a context change following acquisition (ABA) as compared to no context change (AAA) while a forth study did not observe such an effect in US expectancy ratings (Dibbets et al., 2008). Furthermore, when reconciling a study (ABA/ABB) by Milad et al. (2005), larger SCRs for both CSs were evident on the first trial following a context switch after acquisition. As no AAA control group was included, the effect of context switch following acquisition could however not be determined statistically.

In addition, there is suggestive evidence for a different course of extinction learning after a context switch. Vansteenwegen et al. (2005) reported faster and incomplete extinction (as assessed by SCRs) following a context switch (ABA vs. AAA) on a descriptive level while Effting and Kindt (2007) do not find a modulating effect of context in SCRs on either a statistical or descriptive level.

Besides the renewal literature, studies investigating the effect of exposure to multiple contexts during extinction learning on ROF (Shiban et al., 2013; Dunsmoor et al., 2014) may be informative with respect to the effects of contextual change from acquisition to extinction. However, also these studies did not statistically test possible effects of contextual change on extinction learning and conditioned responding during extinction.

Taken together, the preliminary findings from the (renewal) literature concerning the impact of a context switch between acquisition and extinction on extinction learning are difficult to interpret and incomplete. Further complicating matters, different dependent measures may differentially reflect specific aspects of the context switch phenomenon, which calls for a multi-modal approach and a systematic investigation in future studies.

To fill this gap, the current study aimed to test the effects of context change on conditioned responding and extinction learning by comparing a group with and without a context change after acquisition (AA vs. AB) with regard to multiple fear responses (skin conductance, subjective ratings and fear potentiated startle) in a fear-conditioning and extinction paradigm. Thereby, we specifically focus on immediate shifts in and the time-course of conditioned responding during extinction. Thereby, the different dependent measures were employed to capture effects of contextual change on different levels of responding such as the affective level (FPS), general arousal (SCRs), and a more cognitive level (self-reports).

## Materials and methods

### Participants

The study sample included 216 right-handed (as assessed by self-report) healthy individuals. Three participants aborted the experiment, 28 had to be excluded due to either technical problems during data acquisition or electrode misplacement, leaving 185 participants for final analyses. Participants were randomly assigned to one of four experimental groups, one undergoing context change (AB; *n* = 36, 26 females) and the others undergoing no contextual change between conditioning and extinction (AA; *n* = 149, 109 females) (see experimental design for details). The AA group consisted of three experimental groups differing in a post-extinction manipulation, which will be reported elsewhere. The two experimental groups did not differ in age and sex distribution (see Table 1). None of the participants reported a history of psychiatric disorders. Written informed consent in accordance with the Declaration of Helsinki was obtained from each participant, and the Ethical Review Board of the German Psychological Association (DGPS) approved the study. Participants were payed for their participation.

**Table 1:** **Descriptives and statistics of the sample per group**.

	**AA**	**AB**	**Statistics**	***p*-value**
N female/male	109/40	26/10	χ^2^ = 0.01	0.91
Age in years (± SD)	25 ± 4	25 ± 4	^*t*^(181^a^) = 0.05	0.96
Mean US intensity [mV(± SD)]	4.28 ± 4.46	3.79 ± 2.16	^*t*^_(183)_ = 0.65	0.52
STAI state	35.62 ± 8.55	34.94 ± 9.29	^*t*^_(183)_ = 0.42	0.67
Awareness (aware/not aware/uncertain)	96/36/14	27/8/0	χ^2^ = 3.93	0.14

a *Missing data of two participants*.

### Questionnaires

State anxiety, personality traits, and internal and external locus of control were examined by using German versions of the STAI (Spielberger et al., 1983), NEO-FFI (Borkenau and Ostendorf, 1993), and IPC (Levenson, 1974; Krampen, 1985) questionnaires, respectively. The STAI was always completed right before the experiment. Other questionnaires were also completed before the experiment but could be finished after the experiment if required by time management.

### Material—electrotactile stimulus

A train of three 2 ms electrotactile square-waves (ISI: 50 ms) was administered to the dorsal part of the right hand and served as the US, generated by a DS7A electrical stimulator (Digitimer, Welwyn Garden City, UK) and delivered through a platinum pin surface electrode (Specialty Developments, Bexley, UK). Prior to the experiment, US intensity was individually adjusted to a level that was considered as being unpleasant but tolerable (range 0.4–40 mA). A standardized protocol was used to calibrate shock intensity. First, the pain threshold was determined, defined as the value that was clearly sensible but not painful. Next, the US intensity was determined by increasing intensities with on average steps of 0.5 mA and asking participants to rate each electrotactile stimulus on a 10 point scale, with 10 being painful and *not* tolerable. It was aimed at to achieve a rating of the electrotactile stimulus that had a value between 7 and 8. Experimental groups did not differ in final intensities (see Table 1) and there were no significant differences between intensities calibrated by the three experimenters, *p* > 0.23.

### Visual material

Black geometrical shapes served as conditioned stimuli (CS; i.e., an ellipse and a rectangle) which were presented on a background color (blue, purple, green or yellow) that served as context for 6 s (Maren et al., 2013; Lonsdorf et al., 2015). One of these shapes (CS+) co-terminated with the US (100% reinforcement ratio during conditioning), whereas the other shape did not (CS−). The context color remained constant for each participant and experimental phase (see also experimental design).

Allocation of the shapes to the CS+ and CS− and background colors was counterbalanced between individuals, as well as the order in which the CS+/CS− appeared. CS presentations were interleaved with a variable inter trial interval (ITI) of 11.5 ± 1.5 s, consisting of a white cross on a black background. Presentation of all stimuli was controlled using Presentation Software (NeuroBehavioral Systems, Albany California, USA).

### Auditory material

A burst of 95dB(A) white noise (“startle probe”) was presented binaurally via headphones (Sennheiser, Wedemark, Germany) 4 or 5 s post CS-onset in half of the habituation trials, 2/3 of the fear conditioning and extinction trials and 5 or 7 s after ITI onset in 1/3 of all ITIs. The last CS presentations during conditioning as well as the first CS presentations during extinction were always startled.

### Experimental design

**Figure 1:**
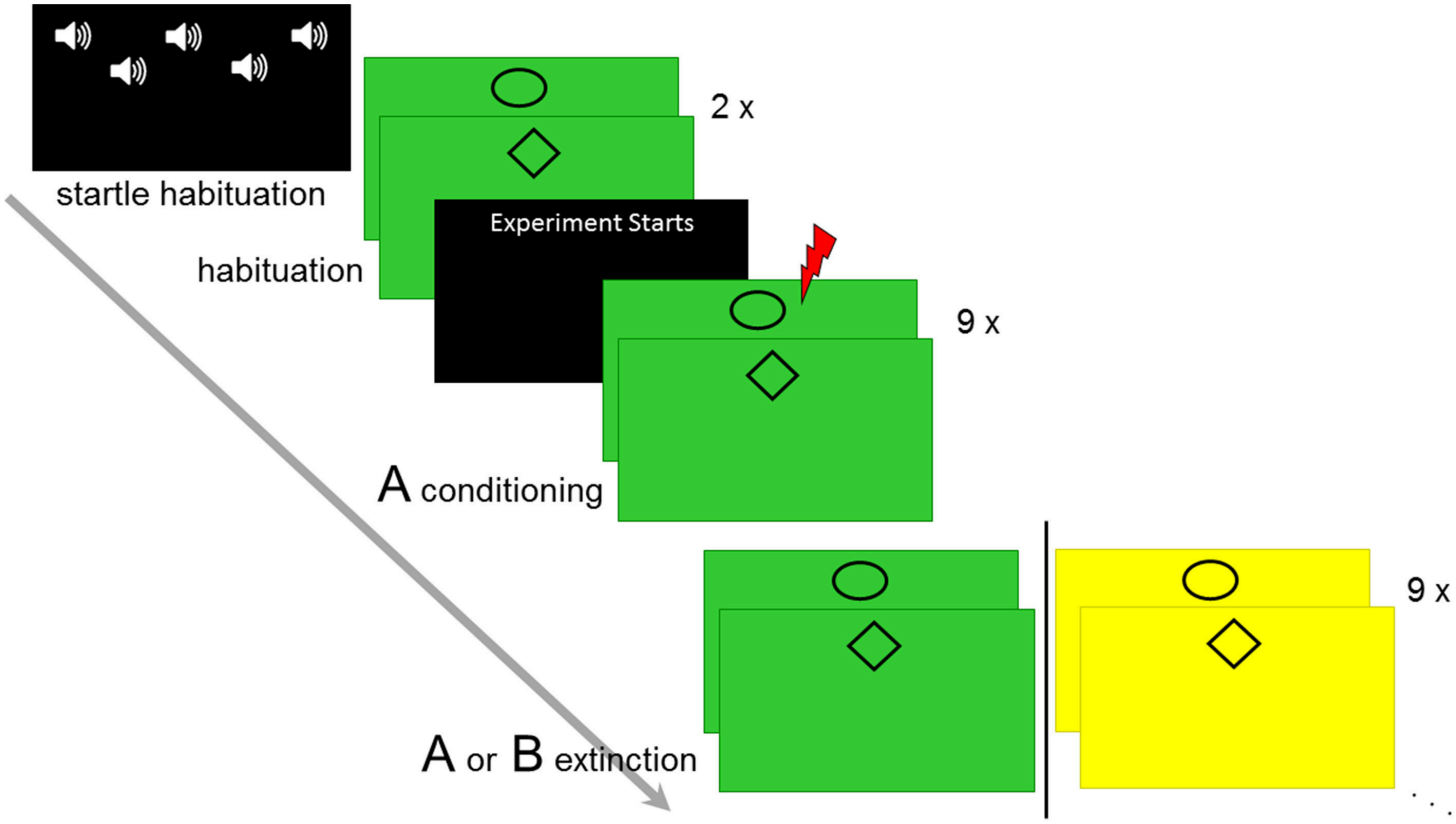
**Overview of the experimental design**. Startle habituation, CS habituation, conditioning in context A, and immediate extinction in context A or B is displayed.

The experiment consisted of seven experimental phases (see Figure 1): US intensity calibration, startle habituation (five startle probes were presented on a black screen to achieve a stable baseline for reactivity), CS habituation (two trials per CS-type, explicitly US-free), conditioning (in context A, nine presentations per CS type), immediate extinction, reinstatement and reinstatement-test. Thereby, extinction took place either in the same context A (AA-group) or a different context B (AB-group) as during conditioning. Reinstatement and reinstatement test differed between participants in contextual allocation and results of this manipulation will be reported elsewhere. Three different female experimenters conducted the experiment.

### Subjective ratings and CS-US awareness

Participants had 7 s to indicate their level of fear, anxiety and distress (“How much stress, fear or anxiety did you experience the last time you saw symbol X?,” with the “X” referring to one of the CS-types at a time) toward both CS-types intermittently throughout the experiment on a visual analog scale (VAS) ranging from 0 (none) to 100 (maximum). The ratings were distributed in a way that they did not indicate the different phases of the paradigm; one was presented at the end of habituation, three during conditioning, three during extinction, and two during reinstatement test. The last rating in the conditioning phase occurred either after conditioning trial 7 or 8 and the first rating in extinction occurred after either extinction trial 1 or 2.

After the experiment participants filled in a post-experimental awareness questionnaire (estimations on the total number of received electrotactile stimuli, questions about CS-US contingencies during the experiment) which were orally confirmed with the experimenter. Based on this, participants were classified as aware, unaware, or uncertain of CS-US contingencies, with the latter in case participants reported a tendency toward the correct contingencies but also unsureness. The number of participants is reduced for rating analyses because some participants failed or were too slow to log their rating during the complete experiment (*n* = 8).

### Physiological parameters—SCR

Physiological data were recorded using a BIOPAC MP100 amplifier (BIOPAC Systems Inc., Goleta, California, USA) and AcqKnowledge 3.9.2 software. Data preprocessing was conducted in Matlab version 2014b (Mathworks, Natick, MA, USA).

For skin conductance responses (SCR), hands were pre-cleaned with warm water, and consecutively two with hydrogel and Ag/AgCl-sensor recording electrodes (Ø 55 mm) were attached to the surface of the left inner hand, i.e., on the distal and proximal hypothenar eminence. SCR data were recorded continuously at 1000 Hz with a gain of 5 μΩ. Data were offline down sampled to 10 Hz and scored semi-automatically using a custom-made program as foot-to-peak (0.9–4.0 s post CS/US onset) according to published guidelines (Boucsein et al., 2012). The absence of a response within this window, or an increase smaller than 0.02 μS, was scored as a zero-response. SCR measurements that showed recording artifacts or excessive baseline activity were discarded and scored as missing values. Raw SCR amplitudes were normalized by using a log transformation, and range corrected (SCR/SCR_CS_or_US_max_) to control for inter individual variability (Lykken and Venables, 1971). Furthermore, data were smoothed within each phase of the experiment by using a local regression function that used weighted linear least squares and a second-degree polynomial model using Matlab. Participants showing more than 2/3 missing SCRs responses toward US presentations (excluding non-reactions) were classified as non-responders and excluded from all SCR analyses (*n* = 4).

### Physiological parameters—FPS

Fear potentiated startle was measured underneath the right eye by using two AG/AgCl electromyogram (EMG) electrodes placed over the orbicularis oculi muscle and one placed on the participants' forehead as a reference. Startle data were sampled with a gain of 5000 at 1000 Hz and band-pass filtered (28–500 Hz) online, rectified and integrated (averaged over 20 samples). Data were scored semi-automatically as foot-to-peak (20–150 ms post startle probe onset) using the same program as for SCRs according to published guidelines (Blumenthal et al., 2005). Blinks up to 50 ms before the startle probe, recording artifacts or excessive baseline activity were scored as missing values. Raw data were T-transformed. Participants showing more than 1/3 zero-responses or missings were excluded from FPS analyses (*n* = 6).

### Statistical analyses

First, a repeated measures ANOVA [mean of CS-type: CS+/CS−] with group (AA, AB) as between subject variable was performed to confirm that participants were successfully conditioned in both groups. Thereby the first trial of each CS-type was excluded from mean calculation, as no conditioning could have possibly taken place.

To test for the immediate impact of contextual change from acquisition to extinction on subjective and physiogical responding a 2 [CS-type: CS+/CS−] × 2 [time: last acquisition/first extinction trial] repeated measures ANOVA with group (AA, AB) as between subject variable was performed on the SCR, FPS, and rating data. Some participants had to be excluded from immediate analyses due to either missing data points on the last acquisition trial or on the first extinction trial (SCR: none; FPS:76; ratings: 32).

Furthermore, to investigate progression of extinction learning two separate repeated measures ANOVAs [CS-type: CS+/CS−] with group (AA, AB) as between subject variable were performed for early and late extinction on physiological and subjective rating data. For physiological measures, early and late extinction was defined as the first half and last half of the trials respectively [SCR: 4 vs. 5, FPS: 3 vs. 3]. Because only three subjective fear ratings were collected during extinction, the first and last rating during the extinction phase defined early and late extinction respectively.

Sex and CS-US awareness were included as covariates of no interest in all analyses, whereas CS-discrimination (difference between CS+ and CS−) was only included as a covariate in time course analyses. A *p*-value of < 0.05 was considered as significant and Greenhouse-Geisser corrected degrees of freedom are reported when appropriate. Partial Eta^2^ (${}_{p}\eta^{2}$) is reported as a measure of effect size. Effects of interest were further tested with additional ANOVA's or univariate analyses. Statistical analyses were performed using IBM SPSS Statistics for Windows, version 22 (Armonk, NY: IBM Corp.). For covariates, only significant or trend-wise main and interaction effects are reported.

## Results

### Manipulation check: Successful fear conditioning

**Figure 2:**
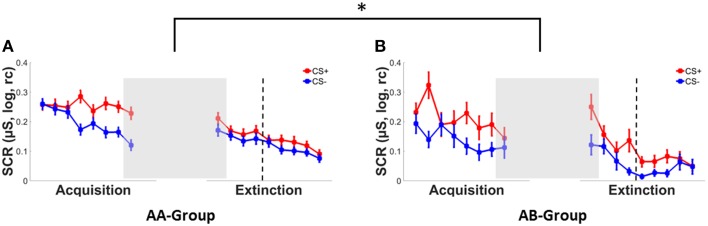
**SCRs to the CS+ (red) and CS− (blue) during conditioning and extinction in (A) a group with both conditioning and extinction in context A (AA) and (B) a group with conditioning in context A and extinction in a new context B (AB)**. Error bars represent the standard error of the mean. Asterisks indicate significant effects with ^*^ indicating *p* < 0.05. Dotted lines separate early extinction from late extinction trials.

Successful fear conditioning was confirmed by a significant main effect of CS-type in SCRs (see Figure 2), ratings (Supplementary Figure 1) and FPS (Supplementary Figure 2), which reflected stronger responses to the CS+ than to the CS−, see Table 2. In addition a main effect of context group was observed for startle responses, with mean responding in the AA group being higher than in the AB group. This indicates pre-existing differences between both groups prior to the experimental manipulation of context change. No main effects of context group were observed for ratings and SCRs (Table 2). In addition, no CS-type^*^context group interactions were observed for any dependent measure.

**Table 2:** **Means and statistics of successful fear conditioning for fear ratings, SCRs and FPS data**.

	**Fear ratings**			**SCRs**			**FPS**		
	**Statistic**	***p*-value**	***p*^η^2^^**	**Statistic**	***p*-value**	***p*^η^2^^**	**Statistic**	***p*-value**	***p*^η^2^^**
CS-type	*F*_(1, 168)_ = 14.68	<0.001	0.08	*F*_(1, 177)_ = 5.32	0.022	0.03	*F*_(1, 171)_ = 18.68	<0.001	0.10
Group	*F*_(1, 168)_ = 0.26	0.613	–	*F*_(1, 177)_ = 2.64	0.106	–	*F*_(1, 171)_ = 6.31	0.013	0.04
Group ^*^ CS-type	*F*_(1, 168)_ = 0.51	0.478	–	*F*_(1, 177)_ = 0.36	0.549	–	*F*_(1, 171)_ = 0.01	0.930	–

#### Effects of covariates (covariates of no interest)

##### Awareness

For SCRs and subjective fear ratings, trend-wise main effects of awareness [SCR: *F*_(1, 177)_ = 2.80, *p* = 0.096, ${}_{p}\eta^{2}=0.02$; ratings: *F*_(1, 168)_ = 3.64, *p* = 0.058, ${}_{p}\eta^{2}=0.02$] as well as trend-wise and significant CS-type^*^awareness interactions were observed [SCRs: *F*_(1, 177)_ = 2.98, *p* = 0.086, ${}_{p}\eta^{2}=0.02$; ratings: *F*_(1, 168)_ = 20.49, *p* < 0.001, ${}_{p}\eta^{2}=0.11$], indicating an expected impact of cognitive contingency awareness on conditioned responding.

### Immediate effect of context switch following acquisition on conditioned responding

#### SCR

An immediate effect of context switch following acquisition (see Figures 2A,B) was evident from a significant CS-type^*^time^*^context interaction in SCRs [*F*_(1, 177)_ = 6.08, *p* = 0.015, ${}_{p}\eta^{2}=0$.03] in absence of any main effects (both *F*'s < 3, *p*'s > 0.10) or two-way interactions (all *F*'s < 1, *p*'s > 0.35).

When testing both CS-types separately, a time^*^context interaction was observed for the CS+ only [*F*_(1, 177)_ = 6.52, *p* = 0.011, ${}_{p}\eta^{2}=0.04$] reflecting increased SCR responding from the last acquisition to the first extinction trial in the AB group (Δ0.11) as compared to the AA group (Δ-0.01) (see Figures 2A,B). No significant main effect of or interactions with context were observed for the CS− (all *F*'s < 1, *p*'s > 0.32).

#### Subjective fear ratings

**Figure 3:**
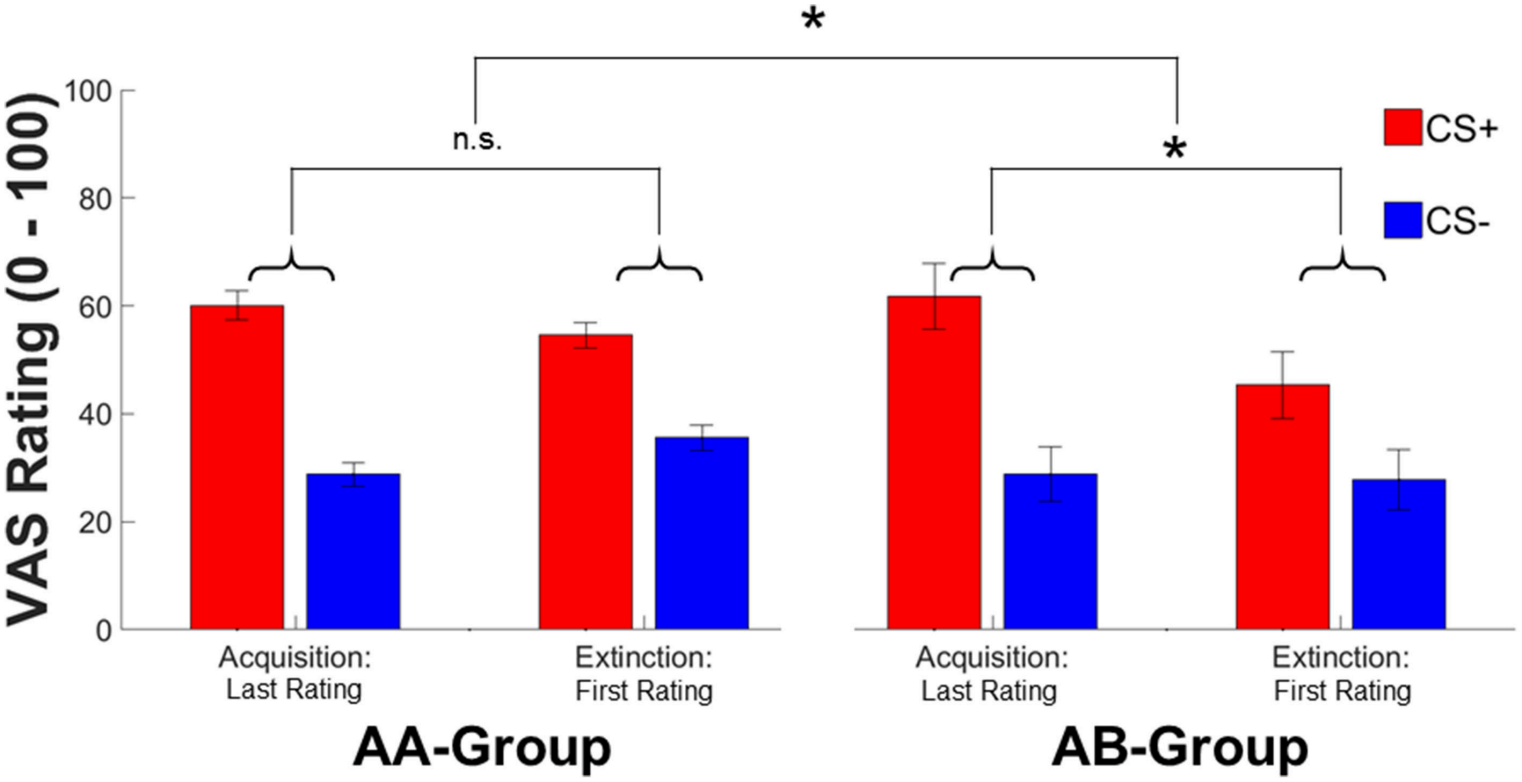
**Mean ratings for the last rating during conditioning and at the first rating during extinction for the CS+ (red) and CS− (blue) in the AA and AB group**. Error bars represent standard error of the mean. Asterisks indicate significant effects between both groups with ^*^ indicating *p* < 0.05.

In contrast to SCRs, subjective fear ratings revealed a time^*^context [*F*_(1, 144)_ = 8.91, *p* = 0.003, ${}_{p}\eta^{2}=0.06$] interaction in absence of a significant CS-type^*^time^*^context interaction (which was significant in SCRs) or CS-type^*^context, and CS-type^*^time interactions (all *F*'s < 2.23, all *p*'s > 0.13). Exploring the time^*^context interaction in more detail revealed that the CS-type independent decrease in fear ratings was conditional to contextual change (AB: Δ-4.77, AA: Δ0.88) (see Figure 3). In addition, significant main effects of CS-type [*F*_(1, 144)_ = 15.25, *p* < 0.001, ${}_{p}\eta^{2}=0.10$; CS+ > CS−] and time [*F*_(1, 144)_ = 4.49, *p* = 0.036, ${}_{p}\eta^{2}=0.03$, conditioning>extinction] were observed in subjective fear ratings.

In sum, both SCR and subjective fear ratings indicate pronounced effects of contextual change on conditioned responding immediately after a context switch. Thereby, the context switch seems to primarily affect CS+ specific responses—at least for SCRs. The absence of a significant effect on subjective ratings might be explained by different times of acquisition for both dependent measures. While SCRs are acquired on a trial-by-trial base, ratings are only acquired intermittently (i.e., after the 1st or 2nd extinction trial). In addition, anticipatory SCR reactions are scored prior to the experience of non-reinforcement during extinction (i.e., CS onset) while ratings are always acquired after the experience of non-reinforcement. As a consequence, rating data might reflect an already partly extinguished phenomenon and thus reflect *reduced* subjective fear after a context switch while SCRs reflect *stronger* responding immediately (1st trial and thus prior to extinction learning) after a context switch.

#### FPS

For FPS (Supplementary Figure 2), in contrast to SCRs and ratings, no main effects or interactions including the factor context, or any other interactions were observed (all *F*'s < 1.50, *p*'s > 0.23) while a trend-wise main effect for CS-type was observed [*F*_(1, 95)_ = 3.12, *p* = 0.081, ${}_{p}\eta^{2}=0.03$; M_cs+_ = 52.96 ± 7.30, M_cs−_ = 49.28 ± 5.76] in absence of a significant main effect of time (*F* < 1, *p* > 0.81).

#### Effects of covariates (covariates of no interest)

##### Awareness

A main effect of awareness was observed in subjective ratings [*F*_(1, 144)_ = 7.03, *p* = 0.009, ${}_{p}\eta^{2}$ = 0.05; uncertain>aware>unaware] while a trend-wise or significant CS-type^*^awareness interaction was observed for SCRs [*F*_(1, 177)_ = 3.38, *p* = 0.067, ${}_{p}\eta^{2}$ = 0.019] and subjective ratings respectively [*F*_(1, 141)_ = 14.80, *p* < 0.001, ${}_{p}\eta^{2}$ = 0.10]. As expected, aware individuals showed stronger CS discrimination than unaware or uncertain individuals during the end of acquisition and at the beginning of extinction in SCRs and subjective fear ratings.

##### Sex

In addition, a trend-wise significant time^*^sex interaction was observed for FPS, *F*_(1, 95)_ = 2.85, *p* = 0.095, ${}_{p}\eta^{2}=0.03$.

### Effects of context switch following acquisition on extinction learning

#### Early extinction—SCR

During early extinction, a significant CS-type^*^context interaction [*F*_(1, 176)_ = 3.90, *p* < 0.050, ${}_{p}\eta^{2}=0.02$] and a trend-wise significant main effect of CS-type [*F*_(1, 176)_ = 2.99, *p* = 0.086, ${}_{p}\eta^{2}=0.02$] were observed in SCRs. Irrespective of contextual change, mean responses tended to be higher for the CS+ (*M* = 0.17 ± 0.17) than for the CS− (*M* = 0.13 ± 0.14). Subsequent univariate analyses showed that mean responses for the CS− were significantly lower in the AB group (*M* = 0.08 ± 0.10) than in the AA group (*M* = 0.15 ± 0.15), but did not differ for the CS+ between context conditions as indicated by a main effect of context for the CS− [*F*_(1, 181)_ = 5.23, *p* = 0.023, ${}_{p}\eta^{2}=0.03$] but not for the CS+ [*F* < 1, *p* > 0.83].

#### Late extinction—SCR

During late extinction however, this CS-type^*^context interaction had vanished [*F* < 1, *p* > 0.47] while a main effect of context was observed, [*F*_(1, 176)_ = 7.05, *p* = 0.009, ${}_{p}\eta^{2}=0.04$], reflecting generally (i.e., CS-unspecific) lower SCR responses in the AB group (*M* = 0.05 ± 0.07) than in the AA group (*M* = 0.11 ± 0.12).

Of note, while a context switch following conditioning lead to immediate (i.e., first trial) CS+ specific *increases* in conditioned SCR responding, effects on the course of extinction learning were CS− specific and reflect a *decrease* in responding in early extinction and a generally (i.e., CS independent) reduction in SCR responding by the end of extinction.

#### Early and late extinction—fear ratings and FPS

In contrast, rating and startle data did not reveal any interactions of or main effects with the factor context and an effect of CS-type was absent in both early and late extinction [both *F*'s < 2.74, both *p*'s > 0.10].

#### Effects of covariates (covariates of no interest)

##### Awareness

For SCRs, a trend-wise CS-type^*^awareness interaction was observed during early [*F*_(1, 176)_ = 3.41, *p* = 0.066, ${}_{p}\eta^{2}=0.02$] but not during late extinction (*F* < 1, *p* > 0.87), while for subjective ratings, a main effect of awareness was observed during early [*F*_(1, 143)_ = 4.47, *p* = 0.036, ${}_{p}\eta^{2}=0.03$, uncertain>aware>unaware] but not late extinction (*F* = 1.78, *p* = 0.184) that was further qualified by a trend-wise CS-type^*^awareness interaction during late extinction only [early: *F* = 0.75, *p* = 0.388; late: *F*_(1, 143)_ = 3.84, *p* < 0.052, ${}_{p}\eta^{2}=0.06$]. This implies that cognitive contingency awareness had an impact on conditioning responding, especially during early extinction.

##### Sex

Furthermore, during both early and late extinction, a main effect of sex was observed in SCRs [early: *F*_(1, 176)_ = 4.13, *p* = 0.044, ${}_{p}\eta^{2}=0.02$; late: *F*_(1, 176)_ = 9.33, *p* = 0.003, ${}_{p}\eta^{2}=0.05$], indicating generally lower SCRs in women than in men. This effect, has been observed previously by our group (Lonsdorf et al., 2015) but an in-depth discussion is beyond the scope of this manuscript. We refer the interested reader to other sources on this topic (Cover et al., 2014; Lonsdorf et al., 2015).

##### CS-discrimination during conditioning

In addition, SCR responding during extinction was significantly affected by the level of CS-discrimination on the last conditioning trial during both early extinction [*F*_(1, 176)_ = 9.22, *p* = 0.003, ${}_{p}\eta^{2}=0.05$] and late extinction [*F*_(1, 176)_ = 6.57, *p* = 0.011, ${}_{p}\eta^{2}=0.04$].

In addition, a significant interaction between CS-discrimination on the last conditioning trial and CS-type was observed for subjective rating data during both extinction phases: *F*_(1, 143)_ = 26.87, *p* < 0.001, ${}_{p}\eta^{2}=0.16$; late: *F*_(1, 143)_ = 18.24, *p* < 0.001, ${}_{p}\eta^{2}=0.11$], indicating that CS discrimination during extinction strongly depends on the discrimination at the end of conditioning.

## Discussion

### Summary

The aim of the current study was to investigate the impact of a contextual change between fear acquisition and extinction on conditioned responding and on the time-course of extinction learning by using a multimodal approach. Generally, our data demonstrate pronounced effects of such contextual change on both immediate conditioned responding and on the time course of extinction learning, which may have important implications for the interpretation of the renewal effect (i.e., effects of contextual switch *after* successful extinction). We report three major findings.

First, immediately after a context switch (i.e., first extinction trial) as compared to no context switch, increased SCRs were observed specifically to the CS+, likely reflecting immediate and intensified conditioned responding. In contrast, subjective fear ratings to both CSs were *attenuated* after contextual change. This apparent discrepancy may be explained by different times of acquisition of SCR and rating data. While ratings were collected *after* one or two extinction trials, SCRs were recorded to each CS *onset*. Hence, the first SCR response following context change is recorded prior to the experience of non-reinforcement (i.e., prior to the possibility of extinction learning), while ratings are provided only after non-reinforcement has been experienced at least once, allowing for extinction to occur. As such, ratings may in fact reflect an already partly extinguished phenomenon while SCR data for the first extinction trial do not.

Second, SCRs to the CS− were attenuated during early extinction (i.e., first half of extinction trials) following a context change. As the effect of early extinction in SCRs, in contrast to the immediate effect, allows extinction to occur, these results line up with lower (albeit in a CS− unspecific way) subjective ratings immediately following a context switch (see above). Consequently, a context change indeed seems to facilitate extinction learning speed for explicit fear ratings to both CS types and specifically to the CS− for the SCRs.

Third, during late extinction (i.e., second half of extinction trials) both CS+ and CS− elicited less SCR responses after a context change following conditioning than without contextual change, indicating not only faster extinction but possibly also more successful extinction (in SCRs) following this context change. However, no difference between both groups were observed in subjective evaluation of the CSs which may reflect a floor effect as subjective ratings had extinguished to floor-level by the end of extinction in both groups.

### Implications

These three major findings highlight that a change in context has a profound differential effect on conditioned responding both immediately following a context change and on the time course of subsequent extinction learning. The direction of this effect however (i.e., facilitated vs. attenuated conditioned responding) is strongly dependent on time (i.e., immediately after contextual change, early and late extinction). While a context switch induced CS+ specific facilitation of conditioned responding in SCRs before extinction learning may take place (i.e., SCRs to the first CS onset), CS− specific facilitated extinction learning in SCRs was observed in subsequent trials. The latter was supported by facilitated extinction learning speed to both CSs in subjective ratings.

Furthermore, our data highlight that single trial analyses in contrast to blocks of averaged trials may reveal divergent findings. These main findings therefore emphasize that the measurement unit (single trial vs. blocked) used for statistical analyses exerts a strong impact on the results which has recently been discussed as an important methodological challenge for ROF research in humans (Haaker et al., 2014). In light of the present data, this may be particularly relevant for ROF studies as both renewal and reinstatement involve contextual changes to varying degrees, and are typically very transient and restricted to one or a few single trials (Haaker et al., 2014).

As studies on extinction and ROF often routinely employ a contextual switch from conditioning to extinction (i.e., AB design), our findings may suggest that the effect of subsequent ROF manipulations may differ from studies employing no contextual change between acquisition and extinction (i.e., AA). First, extinction learning speed was affected by a contextual change from acquisition to extinction, possibly leading to different levels of end-point extinction between AB and AA designs, as shown by our results. Importantly, this end-point extinction responding serves as a baseline to which conditioned responding following ROF is compared to statistically in ROF studies (Haaker et al., 2014). Hence differences in end-point extinction responding are likely to affect the outcome of ROF manipulations.

Second, and perhaps most important, after successful extinction, an inhibitory fear memory trace (extinction memory) is thought to co-exist with the original fear memory trace. Return of fear manipulations are thought to promote recall and expression of this fear memory trace over the extinction memory trace through contextual change (i.e., renewal), which manifests as enhanced (possibly CS+ specific) conditioned responding in the first trial following the contextual change. Our data however demonstrate such a CS+ specific response enhancement following contextual change in absence of the existence of the second inhibitory memory trace. More specifically, CS+ specific response enhancement was induced by contextual change occurring *prior* to extinction learning and thus *prior* to the generation of an inhibitory memory trace. As such, our data suggest that a context switch may exert a general effect on conditioned responding that may -at least partly- also contribute to renewal (and reinstatement) effects.

### Limitations and future directions

Remarkably, in contrast to SCRs and subjective ratings, no effects of contextual change were observed in FPS conditioned responding either immediately after contextual change or during the time-course of extinction. One explanation might be that FPS is a measure of the central nervous system activity (Blumenthal et al., 2005) and indicates fear, whereas electrodermal activity is generally taken as an indication of general arousal (Hamm and Weike, 2005). It might therefore be possible that arousal (SCR) is more sensitive than fear (FPS) to the effects of contextual change. Another possible explanation for the FPS null-finding might be reduced power for FPS data due to numerous missing data points on either the last conditioning or the first extinction trial.

Second, we employed a single-day paradigm with all experimental phases following immediately upon each other. As it has been shown that timing of extinction after conditioning (immediate vs. delayed extinction) affects the course and strength of conditioned responding during extinction (“immediate extinction deficit”) (Maren, 2013), future studies need to investigate whether allowing for memory consolidation in between these phases (acquisition-extinction) in a multiple-day paradigm may result in different findings.

Third, following conditioning, context change and CSs were presented simultaneously and might be perceived a one single compound stimulus. Future studies may thus profit from implementing the context as a constant variable to allow for more clear-cut interpretations or from employing virtual reality techniques (Baas et al., 2004). The latter would contribute to a broader concept/operationalization of context as suggested by Maren et al. (2013) and enhance translational value, as contextual manipulations in rodent work are usually affecting multiple sensory channels.

## Conclusion

In sum, our results demonstrate that a context change between fear conditioning and extinction has a pronounced impact on conditioned responding and on the time-course of extinction learning. As we have demonstrated that the effect of a contextual change on conditioned responding is not exclusively conditional to completed extinction learning (i.e., renewal), our results may challenge the interpretation of mechanisms underlying return of fear induced by renewal. Hence, it is urgent to systematically investigate the role of ROF specific and non-specific effects of contextual change.

## Author contributions

All authors contributed extensively to the work presented in this paper. TL conceived and designed the study, RS and JN acquired, all authors analyzed, and TL and RS interpreted the data. TL and RS jointly drafted the manuscript and JN critically revised the manuscript. All authors give final approval of the version to be published and agree to be accountable for all aspects of the work.

### Conflict of interest statement

The authors declare that the research was conducted in the absence of any commercial or financial relationships that could be construed as a potential conflict of interest.
