# Supplementary figures and images for: Contextual Change After Fear Acquisition Affects Conditioned Responding and the Time Course of Extinction Learning—Implications for Renewal Research
Source: Front Behav Neurosci. 2015 Dec 8;9:337. doi: 10.3389/fnbeh.2015.00337 (PMC4672066; doi:10.3389/fnbeh.2015.00337)

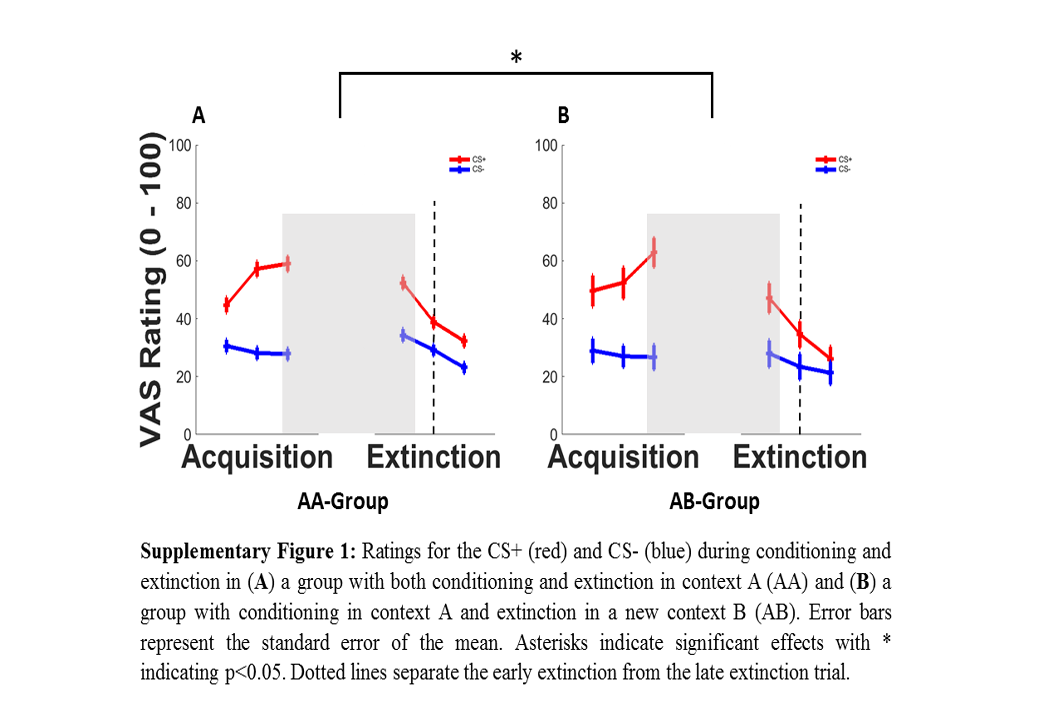

Supplement: Supplementary file 1 [file Image1.TIF]

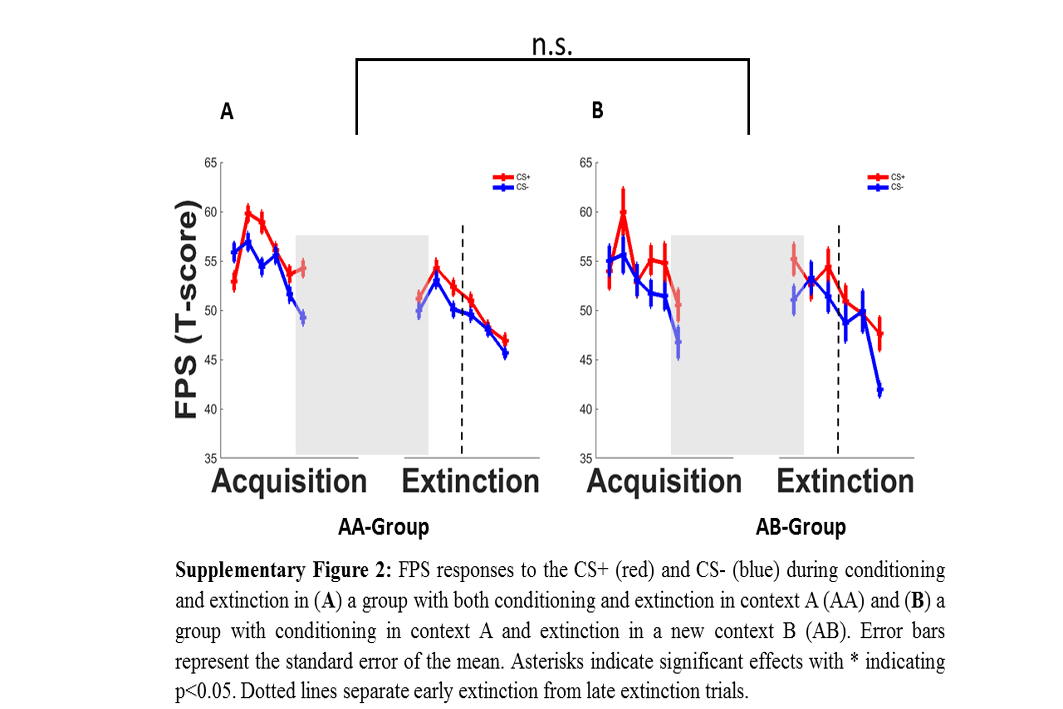

Supplement: Supplementary file 2 [file Image2.TIF]
